# Integrating TBSRTC subcategorization and *BRAF* V600E testing for precision management of Bethesda III thyroid nodules: a WHO 5th edition-based study highlighting subtype-specific diagnostic disparities

**DOI:** 10.3389/fonc.2025.1682593

**Published:** 2026-01-02

**Authors:** Qing Yu, Zhirong Yang

**Affiliations:** Department of Pathology, Deyang People’s Hospital, Deyang, China

**Keywords:** ultrasound-guided fine-needle aspiration, Bethesda system, atypia of undetermined significance (AUS), *BRAF* V600E, papillary thyroid carcinoma, follicular variant, risk stratification, molecular diagnostics

## Abstract

**Objective:**

To investigate the malignant risk of ultrasound-guided fine-needle aspiration (UG-FNA) cytology-diagnosed Bethesda III thyroid nodules and the significance of combined *BRAF* V600E mutation testing, and to establish a clinically actionable algorithm integrating cytomorphologic and molecular features.

**Methods:**

A retrospective analysis was conducted on UG-FNA results from 10,839 patients (12,528 nodules) at Deyang People’s Hospital between December 2021 and September 2024. Liquid-based cytology (LBC) combined with cell block technology identified 732 Bethesda III nodules (detection rate: 5.8%). Among these, 180 cases with postoperative histological follow-up were analyzed, including 101 cases with preoperative *BRAF* V600E testing using cell blocks. Using histology (5th edition WHO classification) as the gold standard, the influence of gender, age, nodule location, diameter, and cytological subcategory (with nuclear atypia vs. with other features) on malignant risk was analyzed using univariate and multivariate logistic regression. The diagnostic value of *BRAF* V600E testing was evaluated.

**Results:**

Among 180 Bethesda III nodules, malignancies accounted for 62.2% (112/180), predominantly papillary thyroid carcinoma (PTC; 110/112, 98.2%), while low-risk neoplasms and benign lesions constituted 15.0% (27/180) and 22.8% (41/180), respectively. Multivariate analysis identified nodule size <1 cm (P < 0.001) and the “nuclear atypia” subcategory as independent predictors, with the latter showing extreme predictive value for malignancy (OR = 121.854, P < 0.001; capturing 98.2% malignancies) and modest association with low-risk neoplasms (OR = 7.014, P = 0.001). *BRAF* V600E testing (n=101) demonstrated 82.0% sensitivity (100% specificity) for PTC but exhibited striking subtype-dependent performance (classical PTC: 90.2% [46/51] vs. FVPTC: 40.0% [4/10], P = 0.001). ROC analysis revealed that combined cytology-*BRAF* testing achieved AUCs of 0.873 (95%CI:0.797-0.948) for malignancy and 0.892 (95%CI:0.822-0.962) for PTC, though accuracy was significantly lower for FVPTC (AUC = 0.481) versus classical PTC (AUC = 0.911, P < 0.001).

**Conclusion:**

In Bethesda III nodules, a diameter <1 cm or the “with nuclear atypia” subcategory indicates a high malignant risk (OR = 121.854), warranting active management. Preoperative *BRAF* V600E testing provides excellent detection for classical PTC (sensitivity 90.2%, specificity 100%) but exhibits limited sensitivity for FVPTC (40%). Based on the 3rd edition TBSRTC and 5th edition WHO classification, our risk-stratified algorithm could reduce unnecessary surgeries by 25-30% in indeterminate nodules while maintaining perfect specificity for classical PTC, achieving optimal clinical decision-making.

## 1 Introduction

The management of Bethesda III thyroid nodules remains a significant challenge in precision thyroidology due to their heterogeneous malignant potential. The Bethesda System for Reporting Thyroid Cytopathology (TBSRTC) classifies these nodules as “Atypia of Undetermined Significance (AUS)” (1), representing a diagnostic “gray zone” where malignancy risk ranges broadly from 10% to 30% in initial reports (2). This uncertainty complicates clinical decisions, as 20-25% of repeated FNAs persist as indeterminate (3), forcing clinicians to choose between potentially unnecessary surgery (lobectomy) or risking a delay in cancer diagnosis.

Recent updates in diagnostic criteria—the 3rd edition TBSRTC (2023) (1) and 5th edition WHO Classification of Endocrine Tumors (2022) (4)—provide critical tools for refinement. The TBSRTC now subcategorizes Bethesda III into *“nuclear atypia”* and *“other”* (e.g., architectural atypia, Hürthle cells) (1), while the WHO recognizes “low-risk neoplasms” (e.g., NIFTP, FT-UMP) as distinct from overt malignancies (4). However, real-world validation of these frameworks for risk stratification, particularly in combination with molecular testing, remains limited.

Although *BRAF* V600E mutation testing is widely used for thyroid nodules (5) —with its clinical relevance underscored by inclusion in authoritative guidelines, even for aggressive cancers, highlighting its central role (6)—the following critical evidence gaps persist in its application to Bethesda III nodules: (i) Subtype-dependent sensitivity disparities between classical PTC and follicular variants lack robust quantification (7);

(ii) The interplay of TBSRTC subcategories with WHO risk stratification remains undefined;

(iii) Evidence-based surgical triage thresholds for high-risk subsets require establishment.

To bridge these gaps, we pursued three translational objectives: First, to validate the 3rd edition TBSRTC subcategories against WHO 5th edition histopathological gold standards; Second, to identify clinicopathological predictors differentiating malignancy from low-risk neoplasms; Third, to quantify *BRAF* V600E’s subtype-specific diagnostic performance (classical PTC vs. FVPTC) for constructing a molecular-enhanced clinical decision algorithm. By addressing these objectives, this study aligns with Frontiers in Oncology’s focus on translating molecular biomarkers into actionable clinical algorithms for early intervention in diagnostically challenging tumors (8).

## 2 Materials and methods

### 2.1 Study population and data collection

This retrospective study was conducted at Deyang People’s Hospital and was approved by the Institutional Review Board/Ethics Committee of our institution (Approval No.: 2025-04-113-K01).

**Figure 1:**
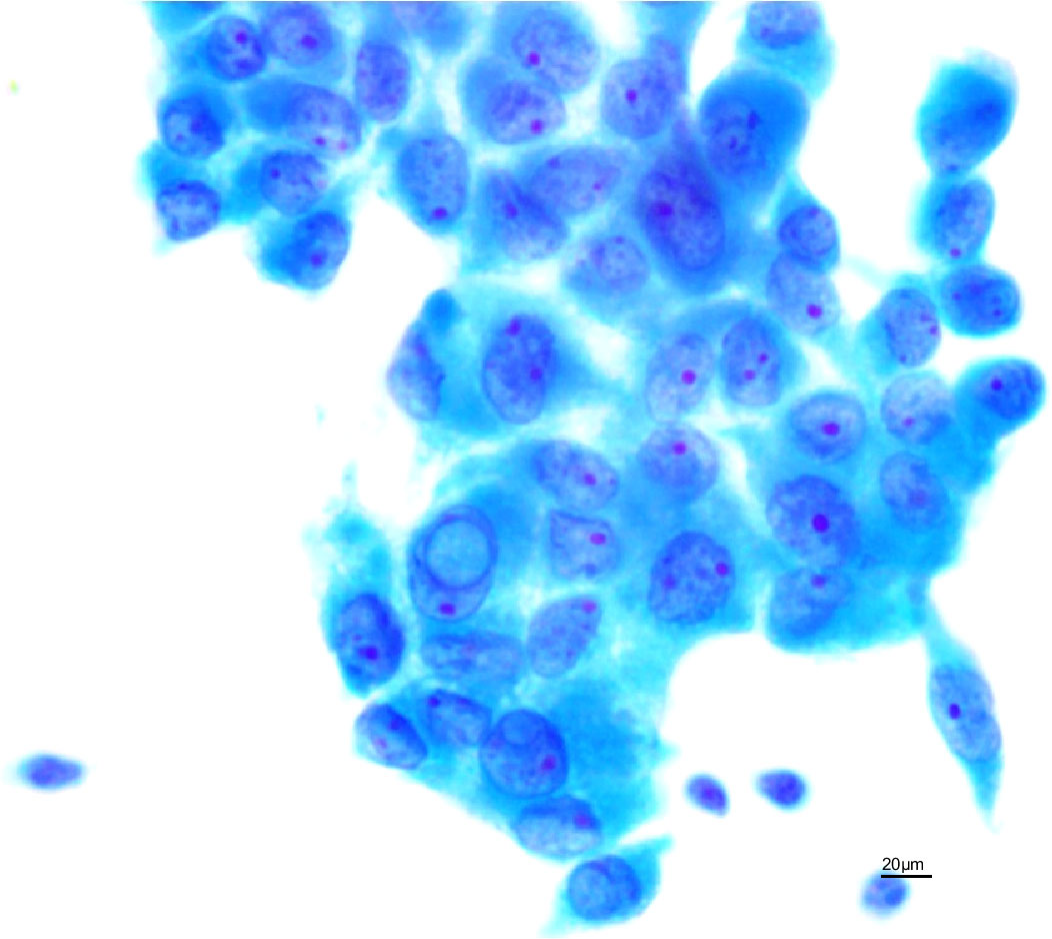
Nuclear atypia (Papanicolaou stain, ×400).

**Figure 2:**
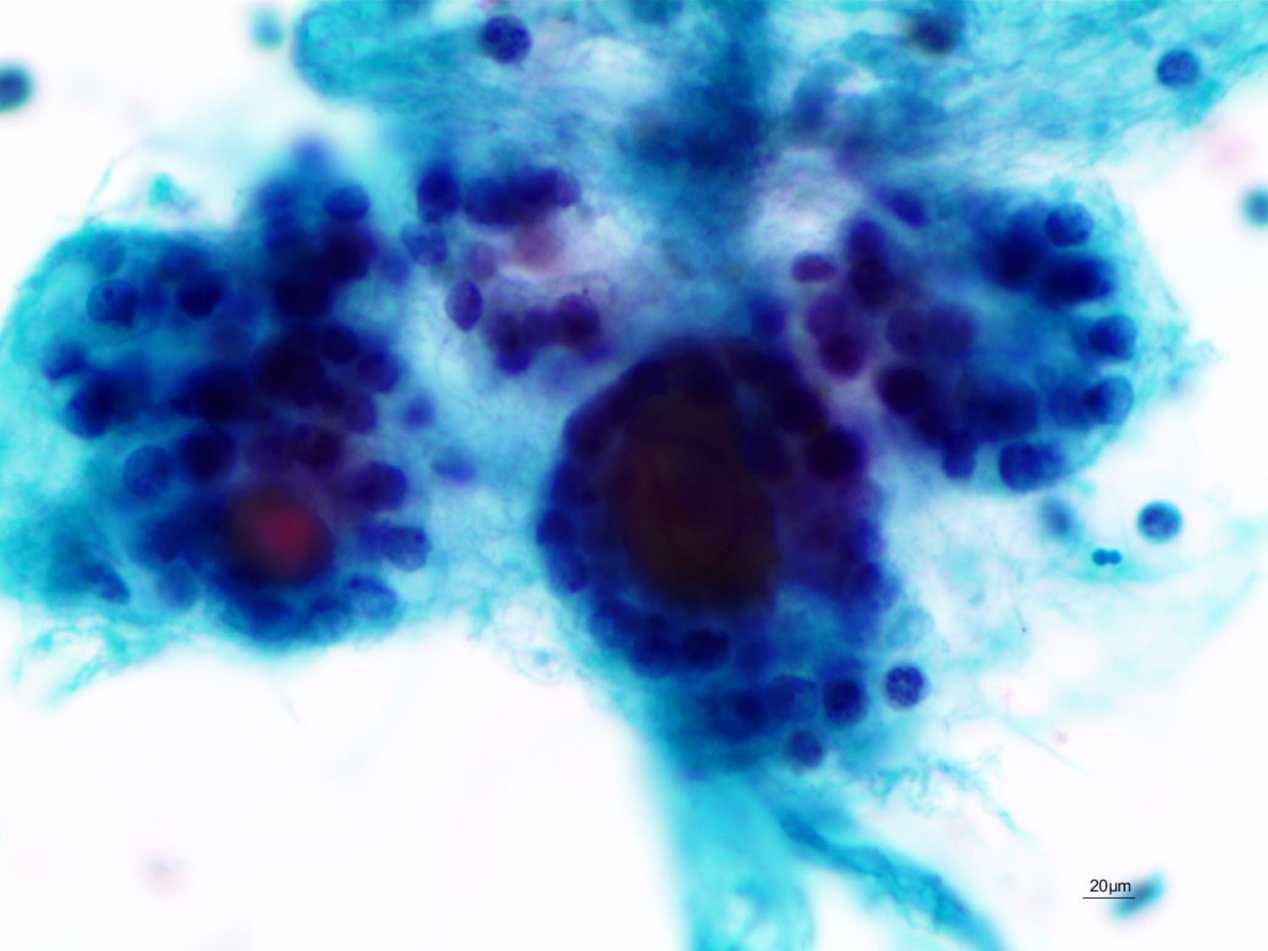
Architectural atypia (Papanicolaou stain, ×400).

Between December 2021 and September 2024, UG-FNA was performed on 12,528 thyroid nodules from 10,839 patients at our institution. Cytological diagnosis followed the 3rd edition of The Bethesda System for Reporting Thyroid Cytopathology (TBSRTC), which identified 732 Bethesda III nodules. These were subcategorized into: “with nuclear atypia” (**Figure 1**) and “with other features” (e.g., architectural atypia, atypical Hürthle cells, atypical lymphocytes) (**Figure 2**). Histological diagnosis adhered to the 5th edition WHO Classification of Endocrine and Neuroendocrine Tumours (2022) (4), categorizing follicular epithelial-derived tumors as benign, low-risk neoplasms, or malignant.

To establish a cohort with definitive histological outcomes, we applied the following inclusion and exclusion criteria to the 732 Bethesda III nodules:

#### 2.1.1 Inclusion criteria

1. Availability of postoperative histological follow-up.
2. Availability of complete clinical and ultrasonographic data.

#### 2.1.2 Exclusion criteria

1. History of previous thyroid surgery, chemotherapy, or radiotherapy.
2. Insufficient cytological material for ancillary studies.

Following this screening process, 180 cases with Bethesda III nodules had postoperative histological follow-up and constituted our final study population. This cohort included 46 males and 134 females, with an age range of 19–77 years (mean ± SD: 45.20 ± 11.82 years). All included patients had complete clinical data and no history of chemotherapy or radiotherapy. Preoperative *BRAF* V600E testing using cell blocks was performed on a subgroup of 101 of these 180 cases, which consisted of 25 males and 76 females, with an age range of 19–77 years (mean ± SD: 44.03 ± 1.17 years).

Data were systematically extracted from our institution’s retrospectively maintained thyroid nodule database and electronic medical records system. The data collection process was performed by two independent researchers to ensure accuracy, with any discrepancies resolved by a third senior investigator.

### 2.2 Clinicopathological characteristics

The following clinicopathological characteristics were meticulously collected for each patient and nodule included in the study:

#### 2.2.1 Patient demographics

Gender and age (categorized as <54 years or ≥55 years).

#### 2.2.2 Nodule characteristics

Location (left lobe, right lobe, or isthmus). Maximum diameter (categorized as ≥1 cm or <1 cm).

#### 2.2.3 Cytological subcategory

Based on TBSRTC, Bethesda III nodules were subcategorized as “with nuclear atypia” or “with other features”.

#### 2.2.4 Histopathological outcome

The final postoperative histological diagnosis, which served as the gold standard, was recorded for all 180 cases.

### 2.3 Cell block preparation and *BRAF* V600E mutation testing

#### 2.3.1 Liquid-based cytology and cell block preparation

Cell blocks were prepared to preserve the residual FNA material for subsequent morphological evaluation and molecular studies, thereby maximizing the diagnostic utility of a single biopsy. Under ultrasound guidance, fine-needle aspiration was performed on thyroid nodules. The aspirated material was immediately rinsed into a 15 mL preservative solution and fixed for at least 30 minutes. The sample was then centrifuged. The supernatant was used for automated liquid-based cytology slide preparation using the BD PrepMate-PrepStain system, followed by Papanicolaou staining for cytological diagnosis.

The residual sediment was collected for cell block preparation. Specifically, the cell pellet was fixed in 10% neutral buffered formalin and subsequently processed through routine dehydration and paraffin embedding according to standard pathological protocols to create formalin-fixed paraffin-embedded cell blocks. Sections of 4-5 μm thickness were cut from the cell blocks. One section was stained with hematoxylin and eosin for morphological evaluation, while consecutive sections were allocated for *BRAF* V600E mutation testing.

#### 2.3.2 DNA extraction and *BRAF* V600E mutation analysis

Genomic DNA was extracted from the FFPE cell block sections using the *BRAF* V600E gene mutation detection kit (AmoyDx, Xiamen, China), strictly following the manufacturer’s instructions. DNA concentration and purity were assessed by measuring the absorbance at A260/A280 ratio using a NanoDrop 2000 spectrophotometer (Thermo Fisher Scientific, USA). Samples with an A260/A280 ratio between 1.8 and 2.0 were deemed to have acceptable quality and were proceeded to downstream mutation analysis.

The *BRAF* V600E mutation was detected using the amplification refractory mutation system polymerase chain reaction (ARMS-PCR) method with the aforementioned commercial kit. The ARMS-PCR method was selected for its high sensitivity in detecting low-frequency mutations in samples with challenging DNA quality, such as those derived from FFPE cell blocks, making it more suitable for this application than less sensitive methods like Sanger sequencing. To ensure assay specificity and sensitivity and to mitigate potential artifacts from FFPE-derived DNA, the following rigorous quality control measures were implemented: (1) Each PCR run included both positive and negative controls provided in the kit to validate the assay’s performance. A no-template control (NTC) was also included in each run to rule out contamination. (2) An internal control gene was co-amplified in each reaction to confirm the integrity of the extracted DNA and the success of the PCR process. (3) Samples with low DNA yield or quality were either re-extracted or excluded from the final analysis. Furthermore, the *BRAF* V600E testing was performed in duplicate for all samples to confirm the reproducibility of the results.

### 2.4 Statistical analysis

All statistical analyses were performed using IBM SPSS Statistics version 25.0. Categorical variables are presented as numbers and percentages, and were compared using the Chi-square (χ²) test or Fisher’s exact test, as appropriate. Continuous variables are presented as mean ± standard deviation or median with interquartile range, and were compared using the Student’s t-test or Mann-Whitney U test, depending on the normality of their distribution, which was assessed using the Shapiro-Wilk test.

The sensitivity, specificity, positive predictive value (PPV), negative predictive value (NPV), and overall accuracy of *BRAF* V600E mutation testing for diagnosing malignancy were calculated with corresponding 95% confidence intervals (CIs). The diagnostic performance of combined models was assessed by receiver operating characteristic (ROC) curve analysis. Differences in the area under the curve (AUC) for diagnosing classical PTC versus follicular variant PTC (FVPTC) were compared using the DeLong test.

To identify independent factors associated with the risk of malignancy, variables with statistical significance in univariate analysis were subsequently included in a multivariate binary logistic regression model. Results are presented as odds ratios (OR) with 95% CIs.

A two-tailed p-value of < 0.05 was considered statistically significant for all tests.

## 3 Results

### 3.1 Histological outcomes of 180 Bethesda III nodules

**Figure 3:**
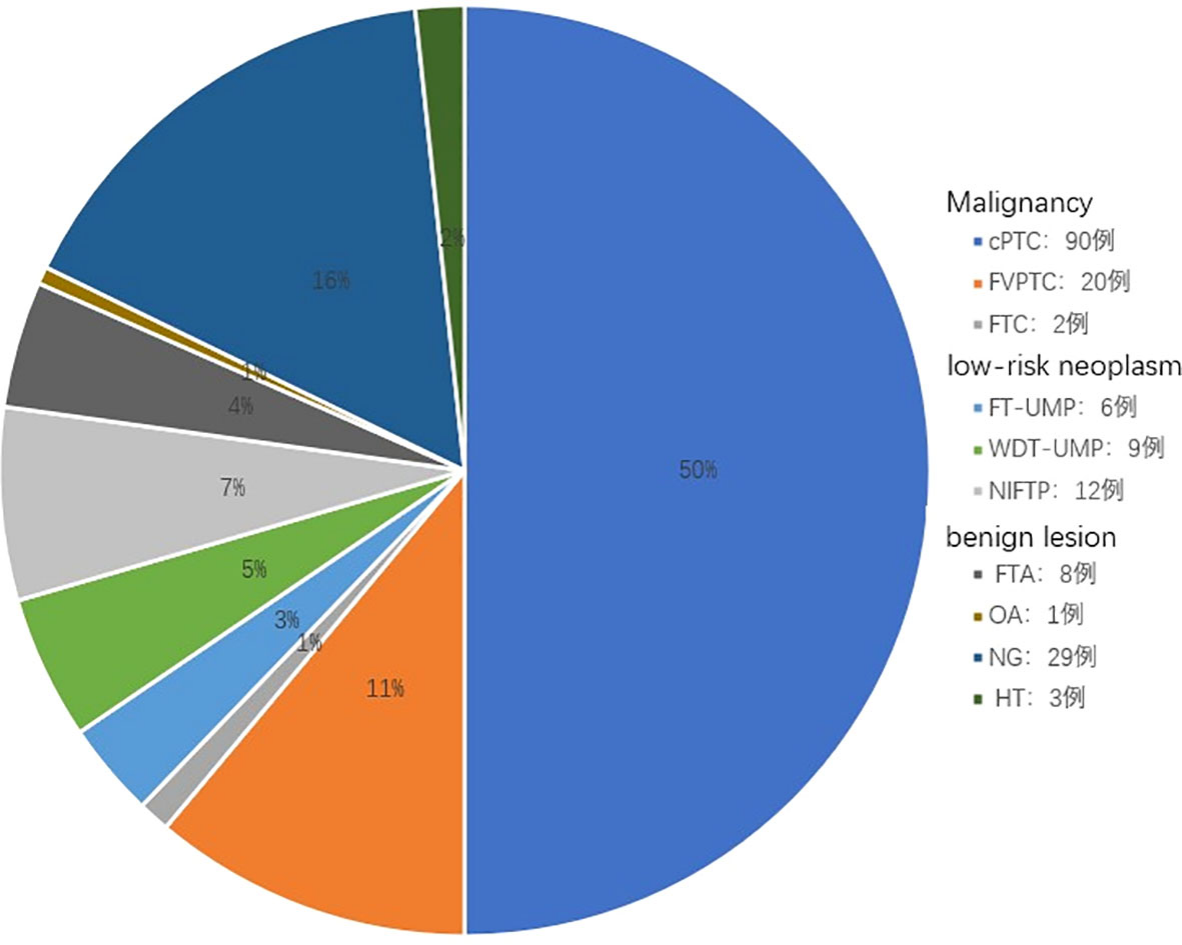
Histological outcomes of 180 Bethesda III nodules.

Among 12,528 nodules from 10,839 patients, 732 Bethesda III nodules were diagnosed using LBC and cell block H&E-stained sections (detection rate: 5.8%, 732/12,528). Postoperative histological follow-up was available for 180 of these 732 nodules: Malignancy rate 62.2% (112/180), low-risk neoplasm rate 15.0% (27/180), and benign lesion rate 22.8% (41/180). See **Figure 3**.

The final diagnosis was based on the 5th edition WHO classification. The cohort comprised 112 malignancies (62.2%), 27 low-risk neoplasms (15.0%), and 41 benign lesions (22.8%). Abbreviations: cPTC, classic papillary thyroid carcinoma; FVPTC, follicular variant of PTC; FTC, follicular thyroid carcinoma; FT-UMP, follicular tumor of uncertain malignant potential; WT-UMP, well-differentiated tumor of uncertain malignant potential; NIFTP, noninvasive follicular thyroid neoplasm with papillary-like nuclear features; FTA, follicular thyroid adenoma; OA, oncocytic adenoma; NG, nodular goiter; HT, Hashimoto’s thyroiditis.

### 3.2 Univariate analysis of clinicopathological features in 180 Bethesda III nodules

Based on postoperative histology, the 180 nodules were divided into Malignancy, Low-risk neoplasm, and Benign lesion groups. Clinicopathological features (gender, age, location, diameter, cytological subcategory) were compared. Significant differences (P < 0.001) were found for nodule diameter and cytological subcategory among the three groups. See **Table 1**.

**Table 1:** Univariate analysis of clinicopathological features in 180 Bethesda III nodules.

Clinicopathological feature	Malignancy	Low-risk neoplasm	Benign lesion	χ² value	P value
Gender [n (%)]				2.829	0.243
Male	24 (21.43)	8 (29.63)	14 (34.15)		
Female	88 (78.57)	19 (70.37)	27 (65.85)		
Age [n (%)]				0.503	0.778
<54 years	88 (78.57)	21 (77.78)	30 (73.17)		
≥55 years	24 (21.43)	6 (22.22)	11 (26.83)		
Location [n (%)]				6.364	0.131
Left	56 (50.00)	9 (33.33)	16 (39.02)		
Right	55 (49.11)	16 (59.26)	24 (58.54)		
Isthmus	1 (0.89)	2 (7.41)	1 (2.44)		
Nodule Diameter [n (%)]				65.999	<0.001
≥1 cm	12 (10.71)	20 (74.07)	27 (65.85)		
<1 cm	100 (89.29)	7 (25.93)	14 (34.15)		
Cytological Subcategory [n (%)]				86.589	<0.001
With other features	2 (1.79)	7 (25.93)	29 (70.73)		
With nuclear atypia	110 (98.21)	20 (74.07)	12 (29.27)		

Values are presented as n (%). P values were calculated by Chi-square or Fisher’s exact test for categorical variables.

### 3.3 Multivariate logistic regression analysis of factors influencing malignant risk in 180 Bethesda III nodules

Using postoperative histology of Bethesda III nodules as the dependent variable (Benign lesion = 0, Low-risk neoplasm = 1, Malignancy = 2), and setting Benign lesion = 0 as the reference group, factors with P<0.05 in univariate analysis (nodule diameter [≥1 cm = 0, <1 cm = 1], cytological subcategory [with other features = 0, with nuclear atypia = 1]) were included as independent variables in the logistic regression model. Results showed that compared to benign lesions: Nodule diameter <1 cm was an independent predictor of malignancy (P < 0.001). The subcategory “with nuclear atypia” was an independent predictor of both malignancy (P < 0.001) and low-risk neoplasms (P = 0.001). See **Table 2**.

**Table 2:** Multivariate logistic regression analysis of factors influencing malignant risk in 180 Bethesda III nodules.

Factor	Regression coefficient	Standard error	Wald χ²	P value	OR (95% CI)
Low-risk neoplasm (Ref: Benign)					
Diameter (Ref: ≥1 cm)	-0.466	0.606	0.591	0.442	0.627 (0.191–2.059)
Subcategory (Ref: Other)	1.948	0.561	12.044	0.001	7.014 (2.335–21.075)
Malignancy (Ref: Benign)					
Diameter (Ref: ≥1 cm)	2.666	0.591	20.361	<0.001	14.387 (4.518–45.809)
Subcategory (Ref: Other)	4.803	0.845	32.299	<0.001	121.854 (23.254–638.539)

ORs were computed using a multivariate logistic regression model based on the WHO 5th edition classification framework, wherein low-risk neoplasms (NIFTP/FT-UMP) were analyzed as an independent histological tier between benign and malignant categories, enabling precise risk estimation for this clinically distinct entity.

### 3.4 Preoperative *BRAF* V600E testing and postoperative histopathology correlation in 101 Bethesda III nodules

**Figure 4:**
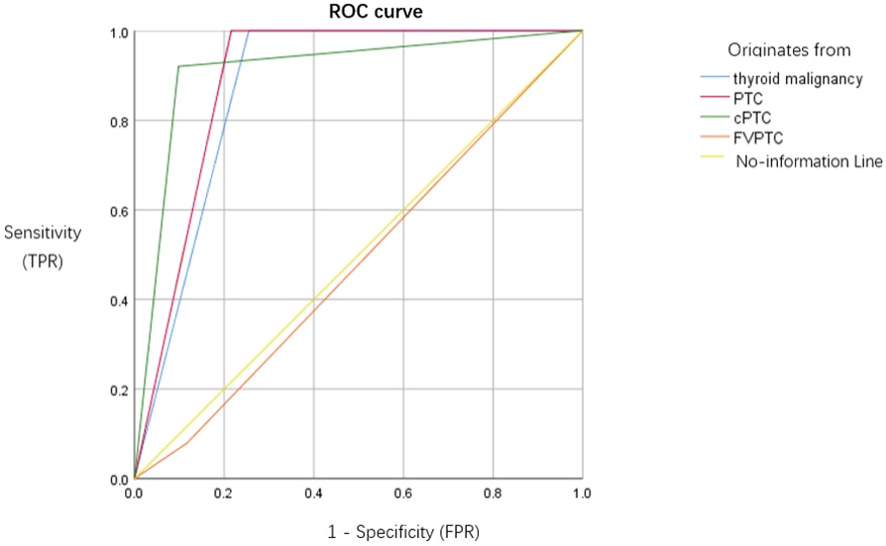
Diagnostic performance of preoperative cytology combined with *BRAF* V600E testing. Receiver operating characteristic (ROC) curves illustrate the combined model’s efficacy in distinguishing thyroid malignancies (blue curve), papillary thyroid carcinoma (PTC) (red curve), classical PTC (cPTC) (green curve), and follicular variant PTC (FVPTC) (orange curve), compared with a no-information line (yellow). The area under the curve (AUC) was significantly higher for classical PTC (AUC = 0.911) than for the follicular variant (AUC = 0.481, P < 0.001 by DeLong test).

Preoperative *BRAF* V600E testing was performed on cell blocks from 101 Bethesda III nodules; mutations were detected in 50 cases. Postoperative histopathology confirmed: 64 malignancies (including 51 classical PTC, 10 follicular Variant of PTC, and 3 other types), 20 low-risk neoplasms, and 18 benign lesions. Among the 50 *BRAF* V600E mutation-positive cases, 46 were classical PTC (92.0%) and 4 were FVPTC(8.0%). ROC analysis showed: Cytology combined with *BRAF* V600E testing yielded an AUC of 0.873 (95%CI: 0.797–0.948) for diagnosing thyroid malignancy and an AUC of 0.892 (95%CI: 0.822–0.962) for diagnosing PTC. Significant subtype differences were observed: AUC was significantly higher for classical PTC (0.911) than for the follicular variant (0.481) (P < 0.001). The sensitivity of the combined test was significantly higher for classical PTC (90.2%, 46/51) than for the follicular variant (40.0%, 4/10) (χ²=14.252, P = 0.001); specificity was 100% for both. See **Figure 4**.

## 4 Discussion

Bethesda III nodules present a significant challenge in thyroid FNA cytology due to inherent diagnostic uncertainty, creating a clinical “gray zone.” The 3rd edition TBSRTC (2023) (1) introduced a crucial update by subcategorizing Bethesda III into “with nuclear atypia” and “with other features (e.g., architectural atypia, Hürthle cells).” This study strictly applied the 3rd edition TBSRTC for cytological diagnosis and utilized the 5th edition WHO classification (2022) (4) for histological triage (benign, low-risk neoplasm, malignant), offering a novel perspective for understanding the biological behavior and enabling precise risk stratification of Bethesda III nodules.

Our study reported a Bethesda III detection rate of 5.8%, strictly within the TBS-recommended range (<10%) (1) and significantly lower than some reports (19.3%–30.6%) (9, 10). This aligns with recent international multicenter studies (e.g., VanderLaan et al., 2023), confirming that standardized diagnostic criteria combined with LBC-cell block technology effectively reduce overuse of the AUS category (11). More importantly, Our malignancy rate (62.22%) far exceeds TBS benchmarks (20-32%) (1). This divergence primarily stems from the high-risk selection bias in our surgically resected cohort (12) and the refined diagnostic criteria of TBSRTC 3rd edition.

### 4.1 Value of TBS subcategorization

The “with nuclear atypia” subcategory, emphasized in the 3rd edition TBS, emerged as a powerful independent predictor of both malignancy (OR = 121.854, P < 0.001) and low-risk neoplasms (OR = 7.014, P = 0.001). Notably, 98.21% (110/112) of malignancies were classified within this subcategory. Our findings robustly demonstrate that this updated TBSRTC criterion significantly improves sensitivity compared to prior reports (78% nuclear atypia-positive rate (13); χ²=15.3, P < 0.001).

### 4.2 Application of WHO classification

The introduction of the NIFTP terminology in 2016 by Nikiforov et al. represented a paradigm shift aimed at reducing the overtreatment of indolent tumors (14), a principle now firmly embedded in the 5th edition WHO classification. By employing this updated framework, we distinctly identified “low-risk neoplasms” (e.g., NIFTP, FT-UMP, WDT-UMP), accounting for 15.0% (27/180) of our Bethesda III cohort. This critical information is often overlooked in studies using a simple benign/malignant dichotomy (15, 16). The accurate identification of these entities is crucial, as their management strategy (often lobectomy alone and de-escalated follow-up) differs significantly from overt malignancies, a point further emphasized in recent updates on NIFTP diagnosis and clinical implications (17). We found that “nuclear atypia” significantly increased the risk of low-risk neoplasms. This finding explains the historical “overestimation” of malignancy risk in older classifications and underscores the clinical importance of the new WHO categories in preventing overtreatment, thereby aligning clinical practice with the original intent of the NIFTP reclassification (14).

### 4.3 Subtype-specific molecular mechanisms

**Figure 5:**
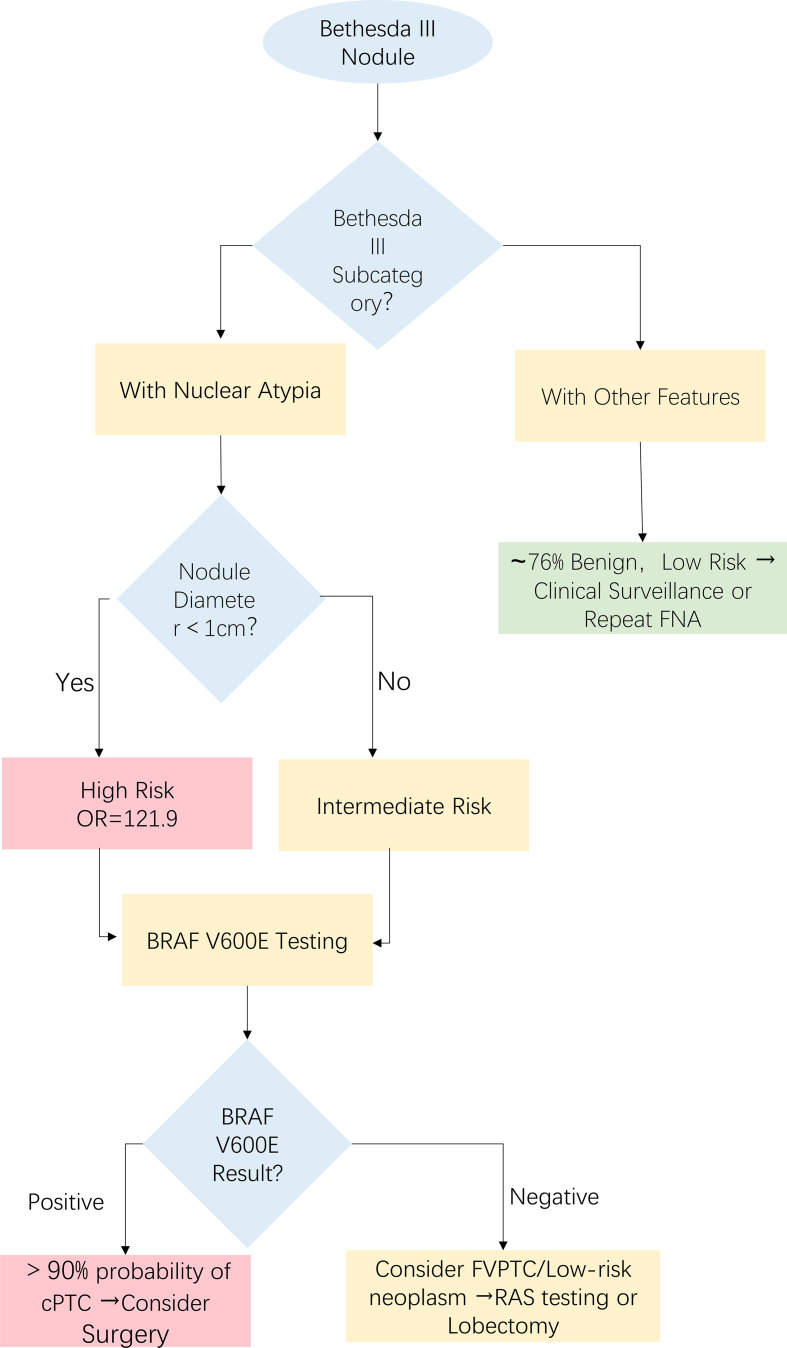
Evidence-based management algorithm for Bethesda III nodules incorporating TBSRTC subcategories and molecular testing. This algorithm integrates TBSRTC subcategorization and *BRAF* V600E mutation testing to guide clinical decision-making. The “with nuclear atypia” subcategory identifies a high-risk cohort. A negative *BRAF* V600E result in this context should raise suspicion for the follicular variant PTC (FVPTC) or other low-risk neoplasms (such as NIFTP), necessitating consideration for supplemental *RAS* testing or lobectomy.

The markedly lower sensitivity of *BRAF* V600E testing for FVPTC (40.0%) compared to classical PTC (90.2%) in our cohort aligns with the established molecular pathogenesis of these subtypes. Our findings provide clinical-diagnostic validation for the fundamental molecular dichotomy, wherein classical PTC is strongly associated with *BRAF* V600E mutations, while FVPTC is predominantly driven by mutations in the *RAS* gene family (18). This divergence underscores a critical diagnostic limitation of a *BRAF*-only approach, which will inevitably miss a significant proportion of FVPTCs and other *RAS*-driven entities like follicular thyroid carcinoma (FTC). Consequently, our clinical algorithm (**Figure 5**) is validated: a negative *BRAF* result in follicular-patterned nodules should prompt consideration for supplemental or expanded molecular testing (e.g., for *RAS*, *TERT*, and *PAX8/PPARγ*), an approach supported by growing evidence on molecular triage for indeterminate nodules (19, 20). Although our FVPTC cohort (n=10) remains modest, it exceeds sample sizes in pivotal studies (Bongiovanni et al. (12): n=8; VanderLaan et al. (13): n=6), and its molecular profile is consistent with larger genomic studies (18), thereby strengthening the evidence for subtype-dependent molecular testing strategies.

### 4.4 Clinical algorithm development

These findings support a stratified clinical approach (**Figure 5**): Nuclear atypia + diameter <1 cm(OR = 121.9) warrants surgical evaluation. *BRAF* V600E+confirms classical PTC (90.2% sensitivity). *BRAF*-negative follicular-patterned nodules require supplemental *RAS/TERT* testing for FVPTC detection (19, 20), consistent with ATA guideline recommendations for molecular triage (21). Importantly, 76.3% (29/38) of other features’nodules were benign, with only 5.3% (2/38) being malignant. Integrating this subcategorization could safely defer immediate surgery in >75% of Bethesda III other features nodules, instead recommending surveillance or repeat FNA, potentially reducing costs by $1,200-$1,800 per patient [based on thyroid lobectomy cost analyses in 7].

### 4.5 Limitations

#### 4.5.1 Selection bias

The retrospective inclusion of surgically resected nodules with suspicious features likely enriched high-risk cases, evidenced by our malignancy rate (62.22%) exceeding general Bethesda III populations (20-30% (1, 12)) though consistent with surgical cohorts (58.7% (12)). Validation in unselected cohorts is essential.

#### 4.5.2 Subtype-specific thresholds

While our FVPTC cohort (n=10) surpasses prior studies (median n=6 (12, 13)), definitive cutoffs require multicenter collaboration through initiatives like the *International Thyroid Tumor Consortium (ITTC)*.

#### 4.5.3 Molecular coverage

Our study was limited to *BRAF* V600E mutation analysis. While this provided excellent specificity and high sensitivity for classical PTC, it does not cover the mutational spectrum of FVPTC, follicular thyroid carcinoma (FTC), and some low-risk neoplasms, which are frequently associated with *RAS* family mutations or, in the case of more aggressive tumors, *TERT* promoter mutations. Consequently, exclusive *BRAF* testing may underestimate the detection of these entities. Furthermore, as our malignancies were predominantly classic PTC, the high sensitivity of *BRAF* V600E testing is most applicable to this subtype. Our findings and the associated algorithm may not be directly generalizable to the diagnostic workup of follicular thyroid carcinoma or oncocytic neoplasms, which are driven by distinct molecular pathways. The incorporation of expanded molecular panels (e.g., including *RAS* and *TERT*) (20) in future studies is therefore critical to improve diagnostic completeness.

#### 4.5.4 Lack of sonographic risk stratification

Our study lacked comprehensive documentation and analysis of sonographic risk stratification (e.g., via TI-RADS or ATA US patterns). The association we observed between smaller nodule size (<1 cm) and higher malignancy risk may be confounded by the fact that such nodules are typically selected for FNA and subsequent surgery based on the presence of suspicious ultrasonographic features. Therefore, the predictive value of nodule size in our study should not be interpreted in isolation from its ultrasonographic context, and this represents an important limitation of our retrospective design.

### 4.6 Future directions

Building upon the limitations of this study, we propose the following specific avenues for future research to advance the precision management of Bethesda III nodules:

#### 4.6.1 Validation of expanded molecular panels

Large-scale, prospective multicenter studies, utilizing unselected cohorts to minimize surgical bias, are crucial to validate the real-world diagnostic performance of expanded molecular panels. This includes commercially available tests (e.g., ThyroSeq (22), Afirma (23)) as well as targeted next-generation sequencing panels for genes including *RAS*, *TERT*, and *PAX8/PPARγ*. A key focus should be to definitively establish the sensitivity and negative predictive value of these panels specifically in *BRAF* V600E-negative cases, which represent the current diagnostic blind spot.

#### 4.6.2 Development of integrated diagnostic models and health economics research

Future studies should aim to develop and validate integrated diagnostic models that combine cytomorphologic subcategorization, molecular profiling, and sonographic risk stratification. Furthermore, the cost-effectiveness of these refined, multi-parameter algorithms must be evaluated to determine whether their implementation leads to a net reduction in unnecessary surgeries and improves patient outcomes, thereby justifying their use from a healthcare resource perspective.

## 5 Conclusion

This study, conducted within the contemporary frameworks of the TBSRTC 3rd edition and WHO 5th classification, validates a refined risk-stratification approach for Bethesda III thyroid nodules. We demonstrate that the “with nuclear atypia” subcategory is a powerful predictor of malignancy, while also carrying a significant association with low-risk neoplasms. This critical distinction underscores the necessity of tailoring surgical management—favoring lobectomy for initial diagnosis to avoid overtreatment—based on the final WHO classification.

Preoperative *BRAF* V600E testing emerges as a highly specific rule-in tool for classical PTC but exhibits limited sensitivity for the follicular variant, highlighting an inherent diagnostic gap that warrants supplemental molecular testing (e.g., for *RAS*) in *BRAF*-negative cases. Furthermore, while subcentimeter size was a risk factor in our cohort, its interpretation likely depends on concomitant sonographic suspicion.

In summary, the integration of TBSRTC subcategorization and selective molecular testing provides a robust clinical algorithm. This pathway optimizes surgical decision-making by prioritizing high-risk nodules for intervention while safely deferring surgery for the majority of “other features” nodules, thereby balancing oncologic safety with the imperative to reduce unnecessary procedures.

## Data Availability

The original data presented in the study are openly available in Figshare at https://doi.org/10.6084/m9.figshare.30827132.
